# VER-Net: a hybrid transfer learning model for lung cancer detection using CT scan images

**DOI:** 10.1186/s12880-024-01238-z

**Published:** 2024-05-24

**Authors:** Anindita Saha, Shahid Mohammad Ganie, Pijush Kanti Dutta Pramanik, Rakesh Kumar Yadav, Saurav Mallik, Zhongming Zhao

**Affiliations:** 1 Department of Computing Science and Engineering, IFTM University, Moradabad, Uttar Pradesh India; 2 AI Research Centre, Department of Analytics, School of Business, Woxsen University, Hyderabad, Telangana, 502345 India; 3 School of Computer Applications and Technology, Galgotias University, Greater Noida, Uttar Pradesh 203201 India; 4 Department of Computer Science & Engineering, MSOET, Maharishi University of Information Technology, Lucknow, Uttar Pradesh India; 5 Department of Environmental Health, Harvard T. H. Chan School of Public Health, Boston, MA USA; 6 Center for Precision Health, McWilliams School of Biomedical Informatics, The University of Texas Health Science Center at Houston, Houston, TX 77030 USA

**Keywords:** Lung cancer detection, CT scan, Transfer learning, Image processing, Stacking, VGG19, EfficientNetB0, ResNet101

## Abstract

**Background:**

Lung cancer is the second most common cancer worldwide, with over two million new cases per year. Early identification would allow healthcare practitioners to handle it more effectively. The advancement of computer-aided detection systems significantly impacted clinical analysis and decision-making on human disease. Towards this, machine learning and deep learning techniques are successfully being applied. Due to several advantages, transfer learning has become popular for disease detection based on image data.

**Methods:**

In this work, we build a novel transfer learning model (VER-Net) by stacking three different transfer learning models to detect lung cancer using lung CT scan images. The model is trained to map the CT scan images with four lung cancer classes. Various measures, such as image preprocessing, data augmentation, and hyperparameter tuning, are taken to improve the efficacy of VER-Net. All the models are trained and evaluated using multiclass classifications chest CT images.

**Results:**

The experimental results confirm that VER-Net outperformed the other eight transfer learning models compared with. VER-Net scored 91%, 92%, 91%, and 91.3% when tested for accuracy, precision, recall, and F1-score, respectively. Compared to the state-of-the-art, VER-Net has better accuracy.

**Conclusion:**

VER-Net is not only effectively used for lung cancer detection but may also be useful for other diseases for which CT scan images are available.

## Introduction

**Fig. 1:**
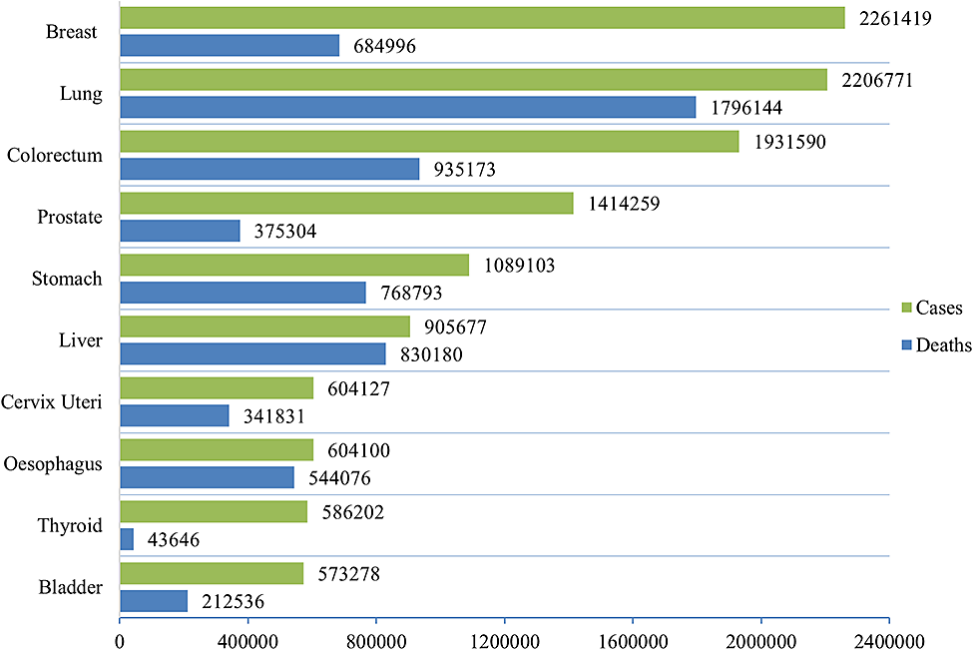
Incident cases and mortality rate of different cancers

Lung cancer is one of the leading causes of cancer-related deaths globally. It is broadly classified as small and non-small-cell lung cancer [1]. Lung cancer is a significant contributor to cancer-related deaths worldwide, with the highest mortality rate among all types of cancer. According to the World Health Organization^1^, cancer is a significant contributor to global mortality, resulting in approximately 10 million fatalities in 2020, which accounts for roughly one out of every six deaths. WHO estimated that one in 16 people would be diagnosed with lung cancer worldwide by 2022. Figure 1 represents the incidence cases and deaths of cancers for both sexes and all age groups worldwide^2^. The x-axis represents the number of people, while the y-axis denotes the types of cancers. Amongst all cancers, lung cancer has a significantly higher mortality rate. Additionally, when considering the number of incident cases, lung cancer ranks second among all types of cancer.

Roughly one-third of cancer-related deaths can be attributed to tobacco usage, a high body mass index, alcohol consumption, inadequate consumption of fruits and vegetables, and a lack of physical activity [2]. In addition, international agencies for cancer research have identified several risk factors that contribute to the development of various cancers, including alcohol, dietary exposures, infections, obesity, radiation, and many more that contribute towards cancer diseases. Lung cancer is caused by the abnormal growth of cells that form a tumour and can have serious consequences if left untreated. Early detection and effective treatment can lead to successful cures for many forms of cancer. Also, it is crucial for improving the survival rate and reducing mortality [3].

Lung cancer is a respiratory illness that affects people of all ages. Symptoms of lung cancer include changes in voice, coughing, chest pain, shortness of breath, weight loss, wheezing, and other painful symptoms [4]. Non-small-cell lung cancer has various subtypes, including Adenocarcinoma, squamous cell cancer, and large cell carcinoma, and is frequently observed [5]. However, small-cell lung cancer spreads faster and is often fatal.

Over the decades, clinical pathways and pathological treatments for lung cancer have included chemotherapy, targeted drugs, and immunotherapy [6]. In hospitals, doctors use different imaging techniques; while chest X-rays are the most cost-effective method of diagnosis, they require skilled radiologists to interpret the images accurately, as these can be complex and may overlap with other lung conditions [7]. Various lung diagnosis methods exist in the medical industry that use CT (computed tomography), isotopes, X-rays, MRI (magnetic resonance imaging), etc. [8, 9].

Manual identification of lung cancer can be a time-consuming process subject to interpretation, causing delays in diagnosis and treatment. Additionally, the severity of the disease infection may not be apparent on X-ray images.

As artificial intelligence (AI) has advanced, deep learning has become increasingly popular in analyzing medical images. Deep learning is a technique that can automatically discover high dimensionality, as compared to the more intuitive visual assessment of images that is often performed by skilled clinicians [10–12]. Convolutional neural networks (CNNs) are promising for extracting more powerful and deeper features from these images [13]. Significant improvements have been achieved in the potential to identify images and extract features inside images due to the development of CNN [14, 15]. Advanced CNNs have been shown to improve the accuracy of predictions significantly. In recent years, the development of computer-aided detection (CAD) has shown promising results in medical image analysis [16, 17]. Deep learning techniques, particularly transfer learning, have emerged as a powerful technique for leveraging pre-trained models and improving the performance of deep learning models [18].

Transfer learning has gained significant attention and success in various fields of AI, including medical image diagnosis [19], computer vision [20], natural language processing [21], speech recognition [22], and many more. Transfer learning involves using pre-trained neural networks to take the knowledge gained from one task (source task) and apply it to a different but related task (target task) [23]. In transfer learning, a model pre-trained on a large dataset for a specific task can be fine-tuned on similar datasets for different tasks.

**Fig. 2:**
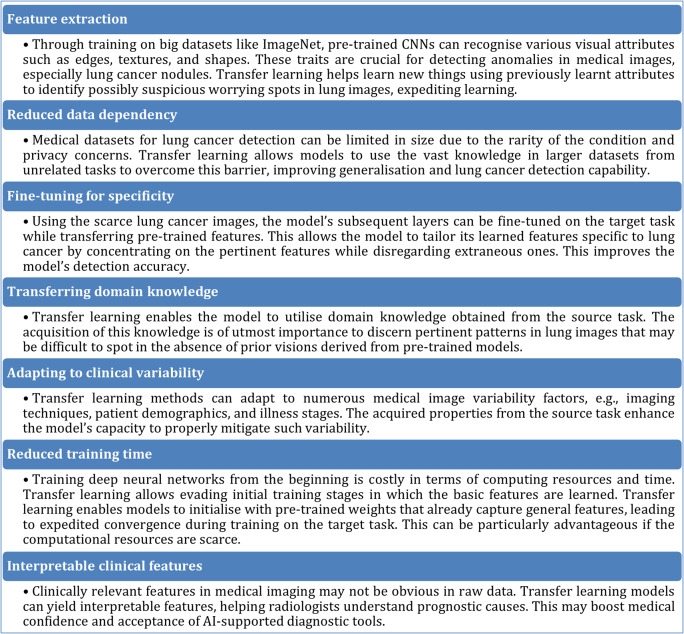
Advantages of transfer learning for lung cancer detection

Transfer learning has recently shown much promise in making it easier to detect lung cancer from medical imaging data. Integrating transfer learning methodologies into pipelines for lung cancer detection has demonstrated enhanced accuracy and efficiency across multiple research investigations. It offers a practical and effective way to leverage existing knowledge and resources to develop accurate and efficient models for lung cancer detection. It starts with a pre-trained CNN model and fine-tunes its layers on a dataset of lung images. This allows the model to quickly learn to identify relevant features associated with lung cancer without requiring extensive labelled lung cancer images. The advantages of transfer learning for lung cancer detection are listed in Fig. 2.

In this paper, we employed different transfer learning models for lung cancer detection using CT images. We proposed a hybrid model to enhance the prediction capability of the pre-trained models. The key contributions of this paper are:

1. The original image dataset is resized into 460 × 460 × 3.
2. Random oversampling is applied to fuse synthetic images in the minority class.
3. Data augmentation is applied by applying shear_range, zoom_range, rotation_range, horizontal_flip, and vertical_flip.
4. Eight transfer learning models, viz. NASNetLarge, Xception, DenseNet201, MobileNet, ResNet101, EfficientNetB0, EfficientNetB4, and VGG19 are tried with the processed dataset.
5. A novel transfer learning model (VER-Net) is built by stacking VGG19, EfficientNetB0, and ResNet101. The outputs of all three models are individually flattened and concatenated afterwards.
6. Seven deep dense layers are added to optimize the performance of VER-Net.
7. The performance of VER-Net is validated on eight different matrices (accuracy, loss, precision, recall, F1-score, macro average, weighted average, and standard deviation) and compared with the other eight considered models.
8. The accuracy of VER-Net is compared with the state-of-the-art.

The rest of the paper is organized as follows. Similar recent research addressing identifying lung cancer through transfer learning is discussed in Sect. 2. The working principle, details of the dataset preparation, and considered transfer learning models are discussed in Sect. 3. Section 4 presents the details of the proposed stacking model, including architecture and parameters. Section 5 presents the proposed model’s experimental details, results, and performance analysis. Section 6 concludes the paper, mentioning the limitations of this study and future scopes.

## Related work

Deep learning techniques provide reliable, consistent, and accurate results. Due to this, they are widely applied across multiple domains to solve real-world problems [24–27]. Researchers have carried out diverse literature that includes datasets, algorithms, and methodology to facilitate future research in the classification and detection of lung cancer. Some of the prominent attempts to detect lung cancer using transfer learning are discussed in this section.

Wang et al. [28] experimented with a novel residual neural network with a transfer learning technique to identify pathology in lung cancer subtypes from medical images for an accurate and reliable diagnosis. The suggested model was pre-trained on the public medical image dataset luna16 and fine-tuned using their intellectual property lung cancer dataset from Shandong Provincial Hospital. Their approach accurately identifies pathological lung cancer from CT scans at 85.71%. Han et al. [29] developed a framework to assess the potential of PET/CT images in distinguishing between different histologic subtypes of non-small cell lung cancer (NSCLC). They evaluated ten feature selection techniques, ten machine learning models, and the VGG16 deep learning algorithm to construct an optimal classification model. The VGG16 achieved the highest accuracy rate of 84.1% among all the models. Vijayan et al. [30] employed three optimizers with six deep learning models. These models included AlexNet, GoogleNet, ResNet, Inception V3, EfficientNet b0, and SqueezeNet. While evaluating the various models, their effectiveness is measured by comparing their results with a stochastic gradient, momentum, Adam, and RMSProp optimization strategies. According to the findings of their study, GoogleNet using Adam as the optimizer achieves an accuracy of 92.08%. Nóbrega et al. [31] developed the classification model using deep transfer learning based on CT scan lung images. Several feature extraction models, including VGG16, VGG19, MobileNet, Xception, InceptionV3, ResNet50, Inception-ResNet-V2, DenseNet169, DenseNet201, NASNetMobile and NASNetLarge, were utilized to analyze the Lung Image Database Consortium and Image Database Resource Initiative (LIDC/IDRI). Among all the algorithms, the CNN-ResNet50 and SVM-RBF (support vector machine– radial basis function) combination was found to be the most effective deep extractor and classifier for identifying lung nodule malignancy in chest CT images, achieving an accuracy of 88.41% and an AUC of 93.19%. The authors have calculated the other performance evaluation matrices to validate the proposed model. Dadgar & Neshat [32] proposed a novel hybrid convolutional deep transfer learning model to detect three common types of lung cancer - Squamous Cell Carcinoma (SCC), Large Cell Carcinoma (LCC), and Adenocarcinoma. The model included several pre-trained deep learning architectures, such as VGG16, ResNet152V2, MobileNetV3 (small and large), InceptionResNetV2, and EfficientNetV2, which were compared and evaluated in combination with fully connected, dropout, and batch-normalization layers, with adjustments made to the hyper-parameters. After preprocessing 1000 CT scans from a public dataset, the best-performing model was identified as InceptionResNetV2 with transfer learning, achieving an accuracy of 91.1%, precision of 84.9%, AUC of 95.8%, and F1-score of 81.5% in classifying three types of lung cancer from normal samples. Worku et al. [33] proposed a denoising first two-path CNN (DFD-Net) for lung cancer detection. During preprocessing, a residual learning denoising model (DR-Net) is used to remove the noise. Then, a two-path convolutional neural network was used to identify lung cancer, with the denoised image from DR-Net as an input. The combined integration of local and global aspects is the main emphasis of the two pathways. Further, the performance of the model was enhanced, and a method other than the traditional feature concatenation techniques was employed, which directly integrated two sets of features from several CNN layers. Also, the authors overcame image label imbalance difficulties and achieved an accuracy of 87.8% for predicting lung cancer. Sari et al. [34] implemented CAD system using deep learning on CT images to classify lung cancer. They used transfer learning and a modified ResNet50 architecture to classify lung cancer images into four categories. The results obtained from this modified architecture show an accuracy of 93.33%, sensitivity of 92.75%, precision of 93.75%, F1-score of 93.25%, and AUC of 0.559. The study found that the modified ResNet50 outperforms the other two architectures, EfficientNetB1 and AlexNet, in accurately classifying lung cancer images into Adenocarcinoma, large carcinoma, normal, and squamous carcinoma categories.

Overall, these studies show that transfer learning has the potential to improve how well medical imaging data can be used to find lung cancer. Using pre-trained deep neural networks can significantly reduce the need for large datasets and reduce training time, making them more accessible for clinical applications. However, more research is needed to find the best architecture for transfer learning and the best fine-tuning strategy for spotting lung cancer. Further studies can focus on improving the interpretability and generalization of transfer learning models for real-world applications.

## Research methodology

The details of the requirements and experimental steps carried out in this paper are discussed in this section.

### Framework

**Fig. 3:**
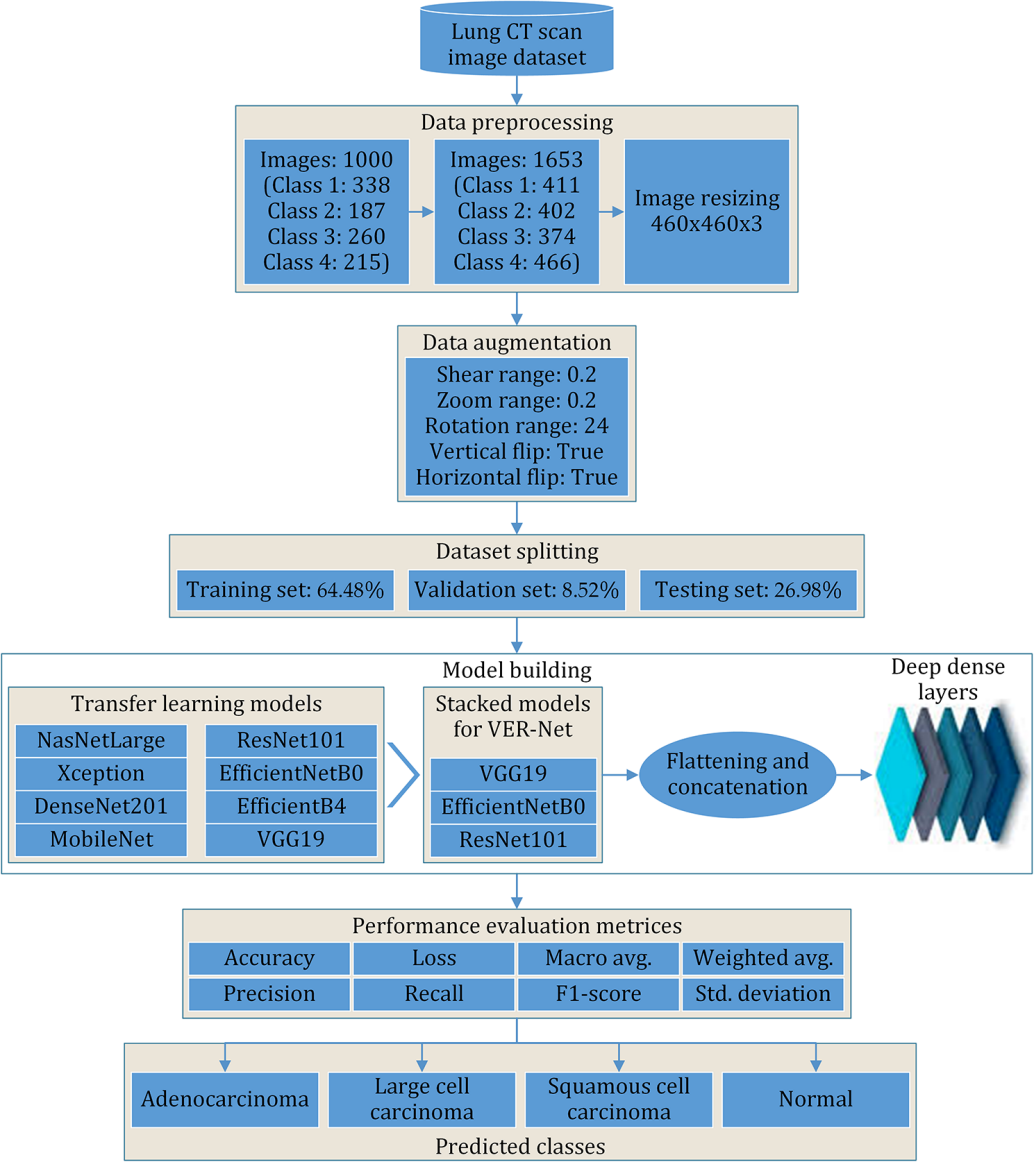
Framework of the proposed methodology

The proposed model follows seven phases of structure, as shown in Fig. 3. After acquiring the chest CT scan images, they were preprocessed and augmented to make the experiment suitable. The processed dataset is divided into training, validation, and testing sets. Eight popular transfer learning models were executed based on this data. Among them, the top three were selected and stacked to build a new prediction model. The model was fine-tuned repeatedly to improve the classification accuracy while reducing the required training time. The model was trained and validated to classify three cancer classes and a normal class. Finally, the model was tested.

### Dataset description

**Fig. 4:**
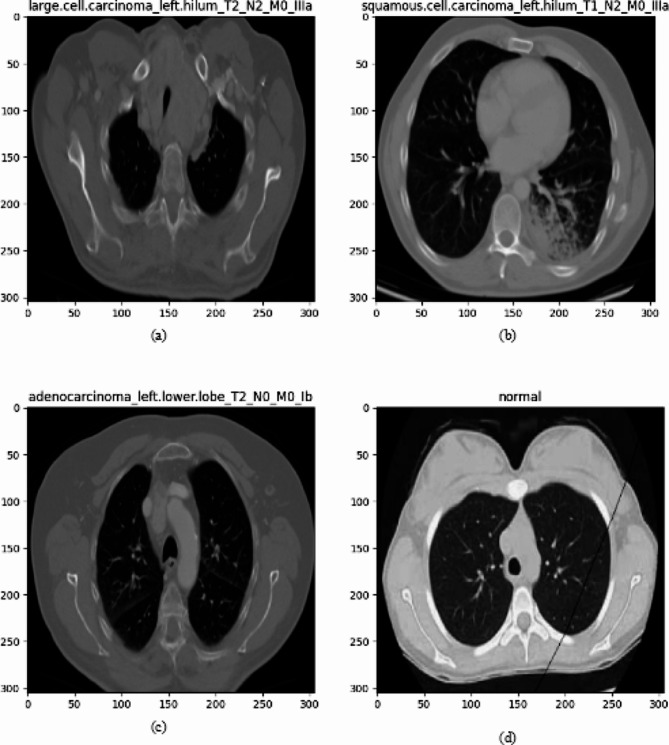
Sample images from chest CT imaging dataset (a) large cell, (b) squamous cell, (c) adenocarcinoma, and (d) normal

The chest CT images utilized in this study were obtained from Kaggle^3^. The dataset contains CT scan images of three types of lung cancers: Adenocarcinoma, Large cell carcinoma, and Squamous cell carcinoma. During the cancer prediction process, the lung cancer image dataset taken from Kaggle consists of 1653 CT images, of which 1066 images are used for training, 446 images for testing and the remaining 141 for validation purposes to determine the efficiency of the cancer prediction system. Class-wise samples of lung cancer CT images are depicted in Fig. 4. The detailed distribution of the dataset in terms of the total images, number of images in each class, number of classes, and labelling in each category is elucidated in Table 1.

**Table 1:** Detailed sample-wise distribution of dataset before resampling

Dataset	Total images	Image properties	Adenocarcinoma	Large cell carcinoma	Normal	Squamous cell carcinoma
Chest CT scan images dataset	1000	No. of images	338	187	260	215
		Class	1	2	3	4
		Label	0	1	2	3

#### Adenocarcinoma

Lung adenocarcinoma^4^ is the most common form of lung cancer, accounting for 30% of all cases and about 40% of all non-small cell lung cancer occurrences. Adenocarcinomas are found in several common cancers, including breast, prostate and colorectal. Adenocarcinomas of the lung are found in the outer region of the lung in glands that secrete mucus and help us breathe. Symptoms include coughing, hoarseness, weight loss and weakness.

#### Large cell carcinoma

Large-cell undifferentiated carcinoma^5^ lung cancer grows and spreads quickly and can be found anywhere in the lung. This type of lung cancer usually accounts for 10 to 15% of all cases. Large-cell undifferentiated carcinoma tends to grow and spread quickly.

#### Squamous cell carcinoma

Squamous cell carcinoma^6^ is found centrally in the lung, where the larger bronchi join the trachea to the lung or in one of the main airway branches. Squamous cell lung cancer is responsible for about 30% of all non-small cell lung cancers and is generally linked to smoking.

The last category is the normal CT scan images.

### Data preprocessing

To develop a robust and reliable automated system, data preprocessing plays a crucial role in the model-building process [35–37]. Preprocessing is an essential step to eliminate the distortions from the images. In this study, data preprocessing, image resizing, and data augmentation were used for better classification and detection of lung cancer, as discussed in the subsections below.

#### Image resizing

The loaded images are standardized and normalized using a standard scaler and min-max scaler as the normalization functions. The files are resized from 224 × 224 to 460 × 460 using a resize function. The classes undergo label encoding, i.e., 0 for class Adenocarcinoma, 1 for class Large cell carcinoma, 2 for class Normal and 3 for class Squamous cell carcinoma.

#### Data augmentation

Random oversampling was applied afterwards to add randomly duplicate examples in the minority class by adding additional images to the classes containing fewer samples in the dataset. Initially, the dataset comprised 1000 images, with each class containing 338, 187, 260 and 215 images. The final dataset after oversampling contains 1653 images, with each class containing 411, 402, 374 and 466 images, as shown in Table 2.

After that, data augmentation was applied by applying shear_range = 0.2, zoom_range = 0.2, rotation_range = 24, horizontal_flip = True, and vertical_flip = True. Finally, the dataset is split into training, testing and validation in 64.48%, 26.98% and 8.52%, respectively. After the preprocessing followed by the Train-test split, the data is fed to models for training.

**Table 2:** Detailed sample-wise distribution of dataset after resampling

Total images	Image properties	Adenocarcinoma	Large cell carcinoma	Normal	Squamous cell carcinoma
1653	No. of images	411	402	374	466
	Class	1	2	3	4
	Label	0	1	2	3

### Transfer learning models

Transfer learning models play a significant role in healthcare for medical image processing [23, 31]. Medical imaging technologies, such as X-rays, CT scans, MRI scans, and histopathology slides, generate vast amounts of visual data that require accurate and efficient analysis. Transfer learning enables the utilization of pre-trained models trained on large datasets from various domains, such as natural images, to tackle medical image processing tasks [28]. The transfer learning models that are considered in this experiment are described in this section.

#### NasNetLarge

Google created the NasNetLarge [38], a neural architecture search network designed for powerful computational resources. This model addresses the issue of crafting an ideal CNN architecture by formulating it as a reinforcement learning challenge. NasNetLarge introduces an approach where a machine assists in designing neural network architecture and constructing a deep neural network without relying on traditional underlying models that concentrate on tensor decomposition or quantization techniques. Notably, NasNetLarge demonstrated exceptional performance in the ImageNet competition, showcasing its state-of-the-art capabilities. The model is tailored to a specific image input size of 331 × 331, which remains fixed and unmodifiable.

The unique advantages of NasNetLarge are:

- Efficient architecture design using neural architecture search.
- Achieves state-of-the-art performance on various image classification tasks.
- Good balance between accuracy and computational efficiency.

#### Xception

The Xception architecture is a popular and strong convolutional neural network through various significant principles, including the convolutional layer, depth-wise separable convolution layer, residual connections, and the inception module [39]. Additionally, the activation function in the CNN architecture plays a crucial role, where the Swish activation function has been introduced to enhance the conventional activation function. The foundation of Xception is rooted in the Inception module, which effectively separates cross-channel correlations and spatial relationships within CNN feature maps, resulting in a fully independent arrangement.

The unique advantages of Xception are:

- Deep and efficient convolutional neural network architecture.
- Achieves high accuracy on image classification tasks.
- Separable convolutions reduce the number of parameters and operations.

#### DenseNet201

DenseNet201 [40] is a CNN with 201 layers. It is based on the DenseNet concept of densely connecting every layer to every other layer in a feedforward manner, which helps improve the flow of information and gradient propagation through the network. It is a part of the DenseNet family of models, designed to address the problem of vanishing gradients in very deep neural networks. The output of densely connected and transition layers can be calculated using Eq. 1 and Eq. 2.

1 $$ {H}_{i}=f({H}_{0},{H}_{1},{H}_{2},{H}_{3}, \dots,{H}_{i-1}) $$

2 $$ {H}_{i+1}=f\left(BN \right({W}_{i+1}\times {H}_{i}\left)\right) $$

where *H*_*i*_ is the output of the current layer, *f* is the activation function, and [*H*_*0*_, *H*_*1*_, *H*_*2*_, *…, H*_*i−1*_] are the outputs of all previous layers concatenated together. Also, *W*_*i+1*_ is the set of weights for the convolutional layer, BN is the batch normalization operation, *f* is the activation function, and *W*_*i+1*_ is the output of the transition layer.

The unique advantages of DenseNet201 are:

- Dense connectivity pattern between layers, allowing for feature reuse.
- Reduces the vanishing gradient problem and encourages feature propagation.
- Achieves high accuracy while using fewer parameters compared to other models.

#### MobileNet

MobileNet [38] is a popular deep neural network architecture designed for mobile and embedded devices with limited computational resources. The architecture is based on a lightweight building block called a MobileNet unit, which consists of a depth-wise separable convolution layer followed by a pointwise convolution layer. The depth-wise separable convolution is a factorized convolution that decomposes a standard convolution into a depth-wise convolution and a pointwise convolution, which reduces the number of parameters and computations. The output of a MobileNet unit and inverted residual block can be calculated using Eq. 3 to Eq. 7.

3 $$ Y=BN\left(\sigma \right({Conv}_{1*1}\left(DW\right(X\left)\right)\left)\right)$$

4 $$ {X}_{in}=X$$

5 $$ X=BN\left(\sigma \right({Conv}_{1*1}\left(DW\right(X\left)\right)\left)\right)$$

6 $$ X=BN\left(\sigma \right({Conv}_{1*1}\left(X\right)\left)\right)$$

7 $$ Y={X}_{in}+X$$

where *X* is the input tensor, DW is the depth-wise convolution operation, *Conv*_*1 × 1*_ is the pointwise convolution operation, σ is the activation function, BN is the batch normalization operation, and *Y* is the output tensor. Also, *X*_*in*_ is the input tensor, *X* is the output tensor of the bottleneck layer, *Conv*_*1 × 1*_ and DW are the pointwise and depthwise convolution operations.

The unique advantages of MobileNet are:

- Specifically designed for mobile and embedded vision applications.
- Lightweight architecture with depth-wise separable convolutions.
- Achieves a good balance of accuracy and model size, making it ideal for resource-constrained environments.

#### ResNet101

Residual Neural Networks (ResNets) are a type of deep learning model that has become increasingly popular in recent years, particularly for computer vision applications. The ResNet101 [41] model allows us to train extremely deep neural networks with 101 layers successfully. It addresses the vanishing gradient problem by using skip connections, which allow the output of one layer to be added to the previous layer’s output. This creates a shortcut that bypasses the intermediate layers, which helps to preserve the gradient and makes it easier to train very deep networks. This model architecture results in a more efficient network for training and provides good performance in terms of accuracy. Mathematically, the residual block can be expressed as given by Eq. 8

8 $$ y=F(x, \left\{{W}_{i}\right\}+x)$$

where *x* is the input to the block, *F* is a set of convolutional layers with weights *W*_*i*_, and *y* is the block output. The skip connection adds the input *x* to the output *y* to produce the final output of the block.

The unique advantages of ResNet101 are:

- Residual connections that mitigate the vanishing gradient problem.
- Permits deeper network architecture without compromising performance.
- It is easy to train and achieves excellent accuracy.

#### EfficientNetB0

EfficientNetB0 [42] is a CNN architecture belonging to the EfficientNet model family. These models are specifically crafted to achieve top-tier performance while maintaining computational efficiency, rendering them suitable for various computer vision tasks. The central concept behind EfficientNet revolves around harmonizing model depth, width, and resolution to attain optimal performance. This is achieved through a compound scaling technique that uniformly adjusts these three dimensions to generate a range of models, with EfficientNetB0 as the baseline. The network comprises 16 blocks, each characterized by its width, determined by the number of channels (filters) in every convolutional layer. The number of channels is adjusted using a scaling coefficient. Additionally, the input image resolution for EfficientNetB0 typically remains fixed at 224 × 224 pixels.

The unique advantages of EfficientNetB0 are:

- Achieve state-of-the-art accuracy on image classification tasks.
- Use a compound scaling method to balance model depth, width, and resolution.
- A more accurate and computationally efficient architecture design.

#### EfficientNetB4

The EfficientB4 [43] neural network, consisting of blocks and segments, has residual units and parallel GPU utilization points. It is a part of the EfficientNet family of models, designed to be more computationally efficient than previous models while achieving state-of-the-art accuracy on various computer vision tasks, including image classification and object detection. The CNN backbone in EfficientNetB4 consists of a series of convolutional blocks, each with a set of operations, including convolution, batch normalization, and activation. The output of each block is fed into the next block as input. The final convolutional block is followed by a set of fully connected layers responsible for classifying the input image. The output of a convolutional block can be calculated using Eq. 9.

9 $$ {y}_{i}=f\left(BN\right({W}_{i}\times {x}_{i-1}\left)\right)$$

where *x*_*i−1*_ is the input to the current block, *W*_*i*_ is the set of weights for the convolutional layer, BN is the batch normalization operation, *f* is the activation function, and *y*_*i*_ is the block output.

Being in the same family, EfficientB4 shares the advantages of EfficientNetB0.

#### VGG19

Visual Geometry Group (VGG) is a traditional CNN architecture. The VGG19 [44] model consists of 19 layers with 16 convolutional layers and three fully connected layers. The max-pooling layers are applied after every two or three convolutional layers. It has achieved high accuracy on various computer vision tasks, including image classification, object detection, and semantic segmentation. One of the main contributions of the VGG19 network is the use of very small convolution filters (3 × 3) in each layer, which allows for deeper architectures to be built with fewer parameters. The output of the convolutional layers can be calculated using Eq. 10.

10 $$ y=f({W}^{*}x+b)$$

where *x* is the input image, *W* is the weight matrix of the convolutional layer, *b* is the bias term, and *f* is the activation function, which is usually a rectified linear unit (ReLU) in VGG19. The output *y* is a feature map that captures the important information from the input image.

The unique advantages of VGG19 are:

- Simple and straightforward architecture.
- Achieves good performance on various computer vision tasks.
- Its simplicity and ease of use make it a favourite among educators.

## Proposed VER-Net model

To find out the best-performing models among the ones discussed in the previous section, we ran them and assessed their performance individually. Among them, VGG19 and EfficientNetB0 were the best performers in all metrics. However, EfficientNetB4 and ResNet101 competed with each other to take the third spot. In some metrics, EfficientNetB4 did better, while in some, ResNet101 was better. Nevertheless, we picked ResNet101 over EfficientNetB4 because it has better testing accuracy and precision, which is crucial for detecting life-threatening diseases like cancer. Therefore, we stacked VGG19, EfficientNetB0, and ResNet101 in our proposed VER-Net model. The complete algorithm for this procedure is shown in Algorithm 1.

**Table:** 

Algorithm 1: Building the VER-Net model
**Input**: Training dataset, validation dataset, test dataset, training epochs, input shape, and batch size.
**Output**: The output is classified into four categories: Adenocarcinoma, Large cell carcinoma, Squamous cell carcinoma and normal class. The model will return the prediction performance metrics.
*// Read data from image folder* 1. data ← data_read (“chest CT images”) *// Perform preprocessing to improve quality assessment of the dataset* 2. preprocessing_function (rotation, width shift, hight shift, shear range, flipping) *//Perform data augmentation to remove the biasness in the dataset* 3. data_augmentaion (resizing, rescaling, padding, random flipping, random rotation) *// Perform data splitting for model building process* 4. data splitting (training set, testing set, validations set, ratio = 64.48:26.98%:8.52 *// Apply pre-trained transfer learning models* 5. NasNetLarge = tf.keras.applications. NasNetLarge (weights = ‘imagenet’, include_top = False, input_shape = None, pooling = None, classifier activation = ‘softmax’) 6. Xception = tf.keras.applications. Xception (weights = ‘imagenet’, include_top = False, input_shape = None, pooling = None, classifier activation = ‘softmax’) 7. DenseNet201 = tf.keras.applications. DenseNet201 (weights = ‘imagenet’, include_top = False, input_shape = None, pooling = None, classifier activation = ‘softmax’) 8. MobileNet = tf.keras.applications. MobileNet (weights = ‘imagenet’, include_top = False, input_shape = None, pooling = None, classifier activation = ‘softmax’) 9. ResNet101 = tf.keras.applications. ResNet101 (weights = ‘imagenet’, include_top = False, input_shape = None, pooling = None, classifier activation = ‘softmax’) 10. EfficientNetB4 = tf.keras.applications. EfficientNetB4 (weights = ‘imagenet’, include_top = False, input_shape = None, pooling = None, classifier activation = ‘softmax’) 11. EfficientNetB0 = tf.keras.applications. EfficientNetB0 (weights = ‘imagenet’, include_top = False, input_shape = None, pooling = None, classifier activation = ‘softmax’) 12. VGG19 = tf.keras.applications. VGG19(weights = ‘imagenet’, include_top = False, input_shape = None, pooling = None, classifier activation = ‘softmax’) *// Combine the top 3 models for the base layer based on their individual performance over the dataset* 13. Stacking_base_models.append (get_model (VGG19 + EfficientNetB0 + ResNet101)) *// Training and validation of the proposed hybrid model* 14. model_hybrid1_Fit (d_train, b, e) ← Train the Stcaked Model (training set) *// Testing of the proposed hybrid model* 15. Result ← model_evaluate *// Performance evaluation metrics of the proposed hybrid model* 16. Accuracy, loss, precision, recall, f1-score, macro avg, weighted avg ← model_hybrid_Evaluate (testing set) 17. return accuracy, loss, precision, recall, f1-score, macro avg, weighted avg

### Model Architecture

**Fig. 5:**
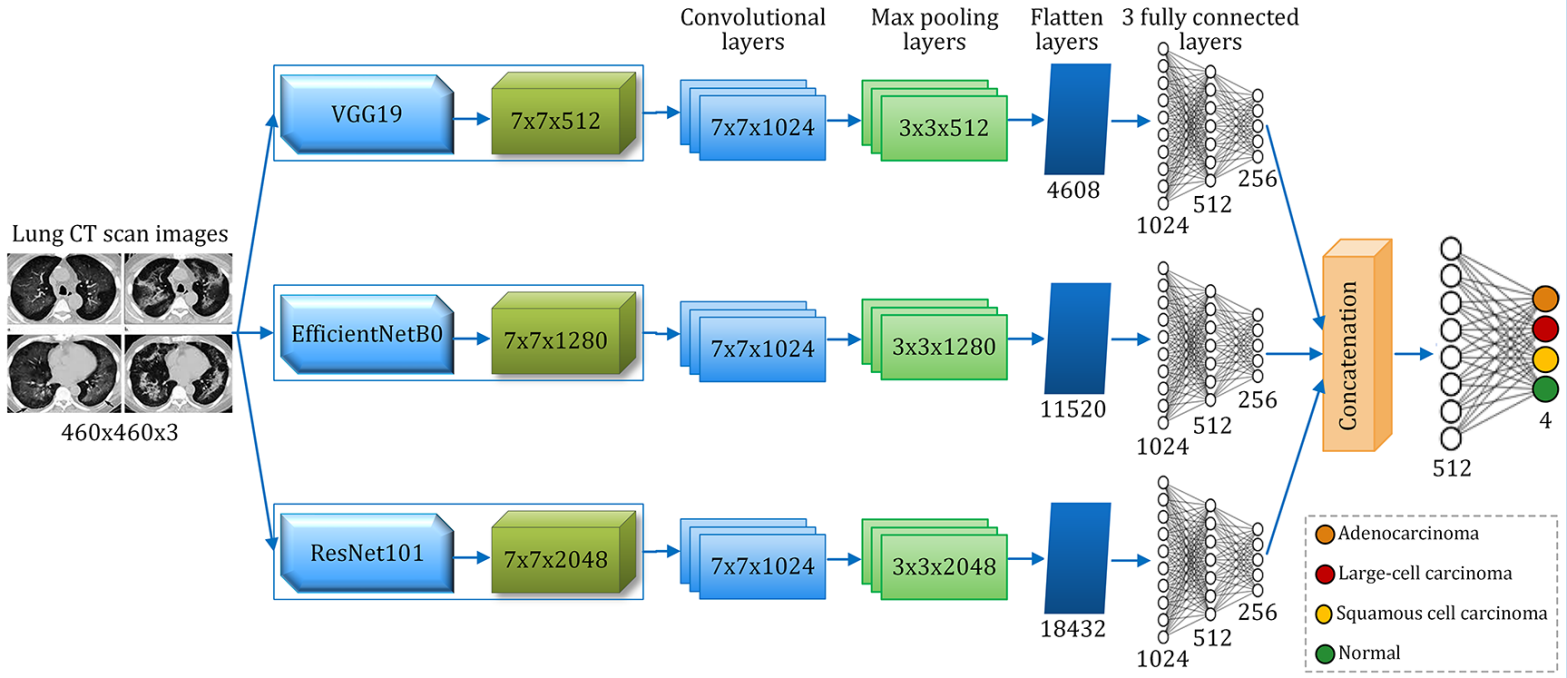
VER-Net’s architecture

The architecture of the proposed VER-Net model is shown in Fig. 5. The input shape is 460 × 460 × 3, which is mapped to four classes as output. We used three different dense layers for three stacked transfer learning models in the model. Thereafter, the same convolution layers of 7 × 7 × 1024 for all three and three different max-pooling layers are used. The outputs are flattened before sending to three 3 fully connected layers (1024 × 512 × 256). The three outputs of these connected layers are then concatenated using majority voting, and accordingly, the classified outputs are generated. The architectural description of VER-Net is shown in Table 3.

**Table 3:** Description of the VER-Net model’s architecture

Layer (type)	Output shape	Param	Connected to
input_3 (InputLayer)	(None, 460, 460, 3)	0	-
modelvgg19(Functional)	(None, 7, 7, 512)	14,714,688	Input_3[0][0][0]
modelefficientnetB0 (Functional)	(None, 7, 7, 1280)	4,049,571	Input_3[0][0][0]
modelresnet101(Functional)	(None, 7, 7, 2048)	18,875,392	Input_3[0][0][0]
flatten (Flatten)	(None, 4608)	0	modelvgg19[0][0][0]
flatten_1 (Flatten)	(None, 11,520)	0	modelefficientnetB0[0][0][0]
flatten _2 (Flatten)	(None, 18,432)	0	modelresenet101[0][0][0]
concatenate (Concatenate)	(None, 33,408)	0	flatten[0][0][0] flatten_1[0][0][0] flatten_2[0][0][0]
dense (Dense)	(None, 1024)	4,719,616	concatenate[0][0][0]
dropout (Dropout)	(None, 1024)	0	dense[0][0][0]
dense_1 (Dense)	(None, 512)	524,800	dropout [0][0][0]
dropout_1 (Dropout)	(None, 512)	0	dense_1[0][0][0]
dense_2 (Dense)	(None, 256)	131,328	dropout [0][0][0]
Dropout_2 (Dropout)	(None, 256)	0	dense_2[0][0][0]

### Model parameters

The details of hyperparameters settings for VER-Net are listed in Table 4. In Table 5, the details of data augmentation are listed. Here, we used the RandomFlip and RandomRotation functions available in TensorFlow.Keras for data augmentation.

**Table 4:** Hyperparameter settings of VER-Net

Hyperparameter	Value
Optimizer 1	Adam
Optimizer 2	RMSprop
Learning rate	0.001
Loss function	Categorical cross-entropy
Dropout probability	0.3
Batch size	32
Number of epochs	100

**Table 5:** Data augmentation details of VER-Net

Particular	Value
Shear range	0.2
Zoom range	0.2
Rotation range	24
Vertical flip	True
Horizontal flip	True
Vertical shearing	0.2
Horizontal shearing	0.2

## Experiment, results and performance analysis

In this section, the experimental details, including system setup and evaluation metrics, are covered. Also, the results are elaborately presented, and the performance of the proposed model is extensively assessed.

### Experimental system setup

The experiment was conducted on a Dell workstation with a Microsoft Windows environment. Python was used to program on the Anaconda framework. The details of the system are given in Table 6.

**Table 6:** System’s hardware and software specifications for the experiment

Component	Specification
Processor	Intel(R) Core(TM)- i9-10900 K CPU @3.70 GHz
GPU	NVIDIA GeForce RTX 2080 TI (11 GB DDR6)
RAM	64 GB (DDR4)
SSD	500GB (NVMe)
Hard Disk	2 TB (HDD)
Operating System	Windows 11 Pro
Platform (IDE)	Anaconda-Jupyter Notebook
Programming Language	Python

### Evaluation Metrics

Evaluation metrics are used to assess the performance of a model on a problem statement. Different evaluation metrics are used depending on the problem type and the data’s nature. In this study, the experimental findings for the presented models are evaluated using various performance metrics, summarised in Table 7.

**Table 7:** Performance evaluation metrics

Metrics	Calculation	Description
Accuracy	$\frac{TP + TN}{TP + TN + FP + FN}$	Gives the classification success of the model, where *TP, TN, FP* and *FN* denote true positives, true negatives, false positives, and false negatives.
Precision	$\frac{TP}{TP + FP}$	Gives a positive estimate value.
Recall	$\frac{TP}{TP + FN}$	Gives the number of positive estimates that are correctly classified.
F1-score	$\frac{2\times TP}{2\times TP + FP+ FN}$	Relates the sensitivity and precision measures.
Loss	$\frac{1}{n}\times \sum _{i=1}^{n}\sum _{c=1}^{k}\left[{y}_{ic}\times \text{log}\left({y}_{ic}\right)\right]$	Measures how well a model is performing by comparing its predictions with the actual target values, where *n* is the number of samples (4), *k* is the number of classes (4), *y*_*ij*_ is the true label (one-hot encoded), and *ŷ*_*ij*_ is the predicted probability for class *c*.
Macro average	$\frac{1}{4}\sum _{c=0}^{3}{A}_{c}^{m}$	The arithmetic mean of the individual class for precision, recall, and f1-score, where *c* denotes classes 0 to 3 and *m* denotes either precision or recall or F1-score.
Weighted average	$$ \sum _{c=0}^{3}{w}_{c}^{m}\times \frac{1}{4}\sum _{c=0}^{3}{A}_{c}^{m}$$	The arithmetic mean of the individual class multiplied by respective weights for precision, recall, and F1-score, where $$ {w}_{0}+{w}_{1}+{w}_{2}+{w}_{3}=1$$.
Standard deviation	$\sqrt{\frac{\sum _{c=0}^{3}{{(S}_{c}^{m}-\frac{1}{4}\sum _{c=0}^{3}{A}_{c}^{m})}^{2}}{n}}$	Deviation of the values or data from an average mean for precision, recall, and F1-score, where *n* is the number of samples (4).

### VER-Net model implementation

After background and designing the VER-Net model, we implemented it. The results are discussed in the following.

#### Confusion matrix

**Fig. 6:**
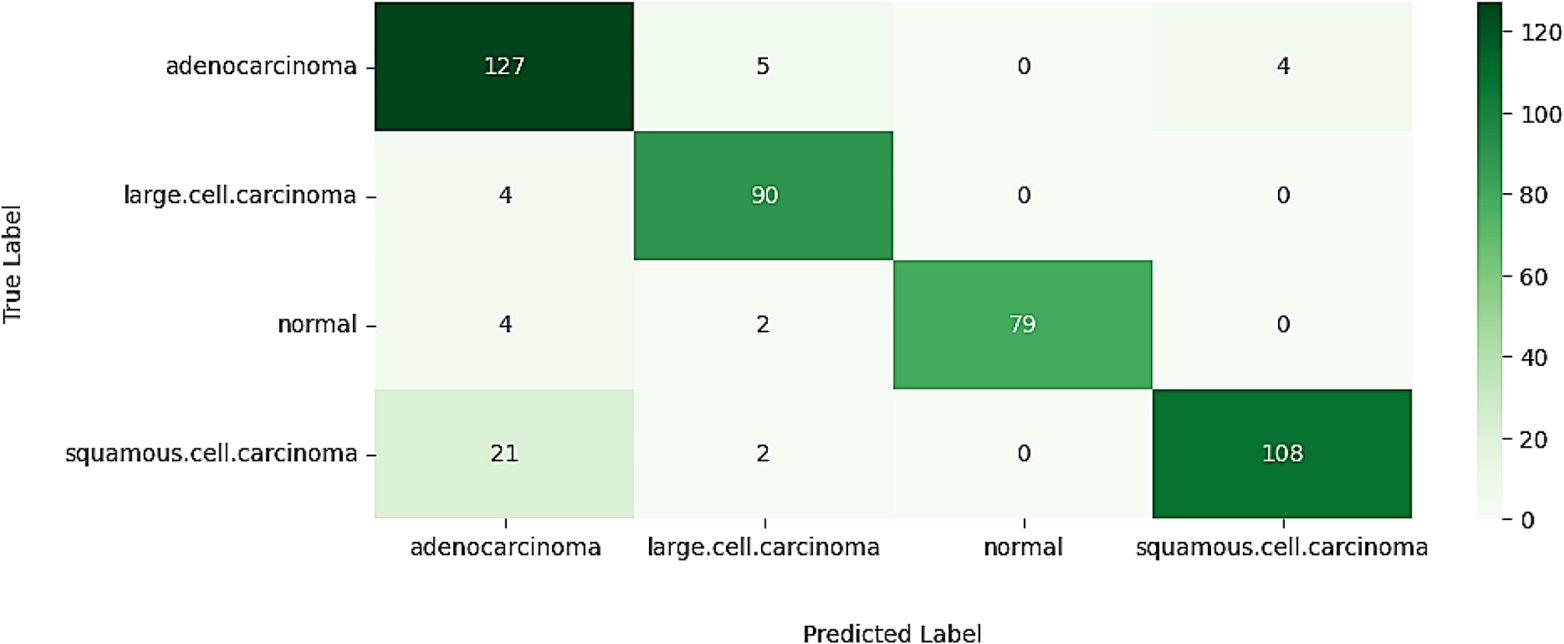
Confusion matrix of VER-Net

The classification performance of VER-Net is evaluated using a confusion matrix, as shown in Fig. 6. Since there are four output classes, the confusion matrix is a 4 × 4 matrix. Every column in the matrix represents a predicted class, whereas every row represents an actual class. The principal diagonal cells denote the respective classes’ correct predictions (TP). Besides the TP cell, all other cells in the same row denote TN. For example, in the first row, except the first column, five of the Adenocarcinoma were falsely classified as large cell carcinoma, and four were categorized as Squamous cell carcinoma. So, 9 (5 + 0 + 4) are TN classifications for the Adenocarcinoma class. Similarly, all other cells in the same column denote FP besides the TP cell. For example, in the first column, except the first row, four Large cell carcinoma, four normal cells, and 21 Squamous cell carcinoma are falsely classified as Adenocarcinoma. So, 29 (4 + 4 + 21) FN classifications exist for the Adenocarcinoma class. The rest of the cells denote FN predictions.

#### Accuracy and loss of VER-Net

**Fig. 7:**
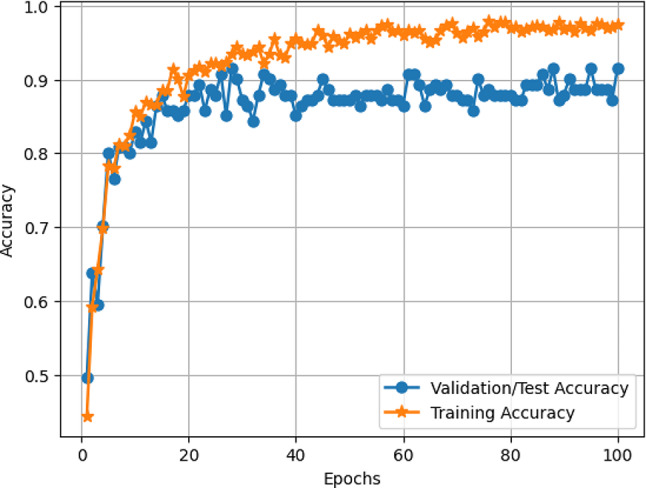
Training and validation/test accuracy VER-Net model

**Fig. 8:**
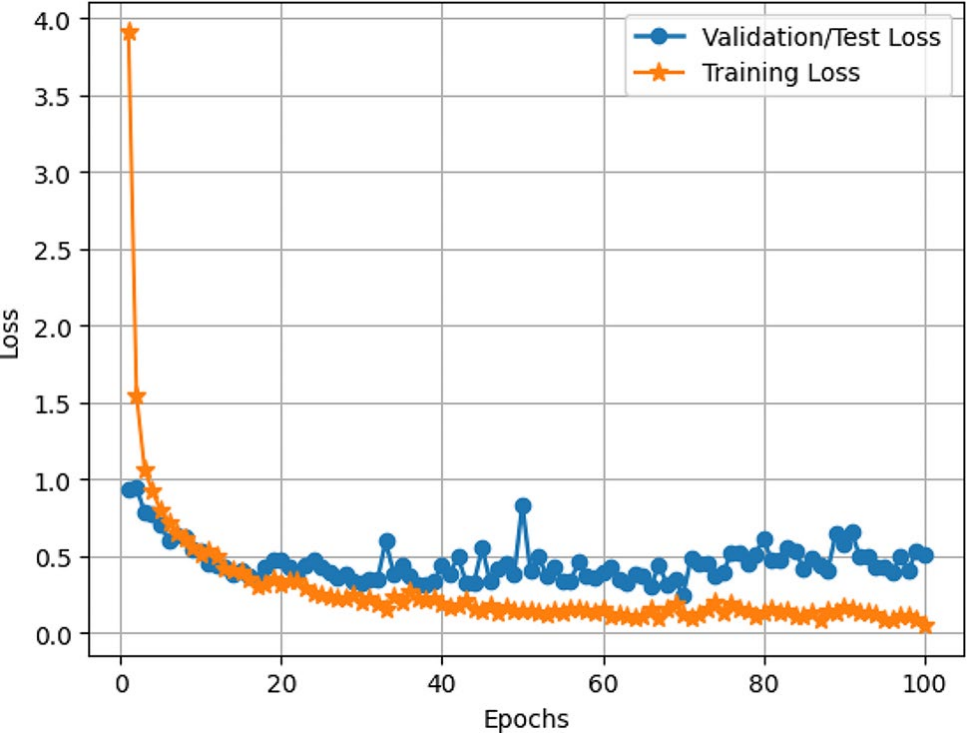
Training and validation/test loss VER-Net model

The accuracy and loss of our VER-Net model are plotted in Figs. 7 and 8, respectively. The x-axis denotes the number of epochs (100), while the y-axis reflects accuracy in Fig. 7 and loss in Fig. 8. The training curve suggests how well VER-Net is trained. It can be observed that both accuracy and loss for validation/testing converge approximately after 20 epochs. It is further noticed that the model did not exhibit significant underfitting and overfitting upon hyperparameter tuning. In our experiment, we tried with different epoch numbers (40, 60, 100, and 200). We got the best results with 100 epochs.

### Performance analysis of VER-Net

In this section, we exhaustively analyze the performance of VER-Net model. For this, we adopted a comparative analysis approach. We compared VER-Net with other transfer learning models and the results of similar research works.

#### Comparing VER-Net with other transfer learning models

First, we compare the performance of VER-Net with the individual transfer learning models, mentioned in Sect. 3.4. All the models were trained and tested on the same dataset and validated with the same parameters.

**Fig. 9:**
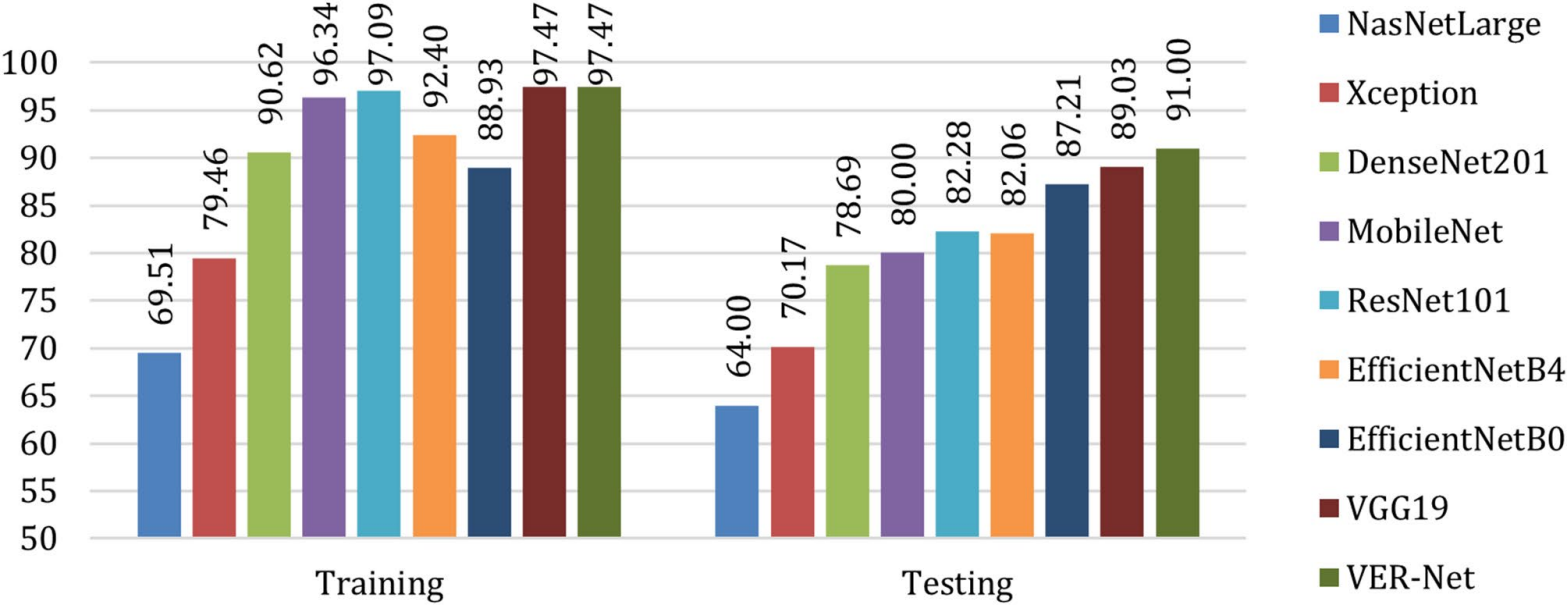
Accuracy comparison of the proposed ensemble method (VER-Net) with other transfer learning models

**Fig. 10:**
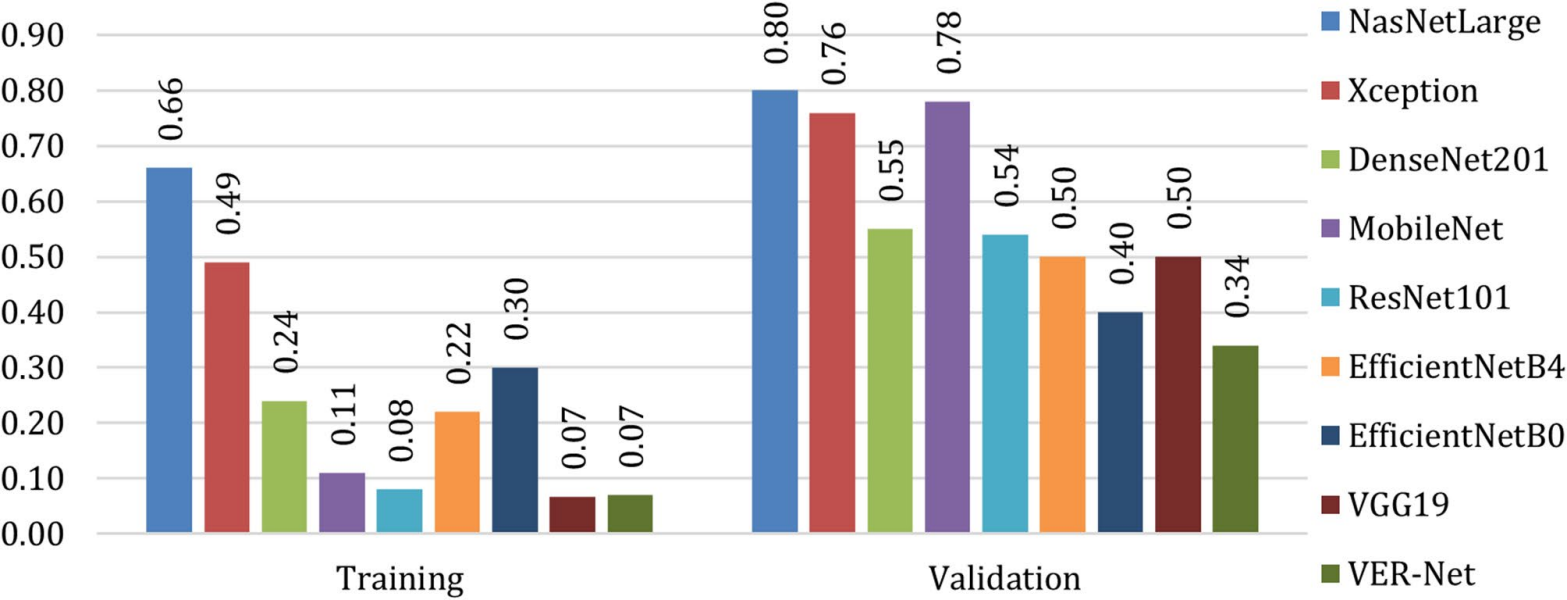
Loss comparison of the proposed ensemble method (VER-Net) with other transfer learning models

Figures 9 and 10 present the accuracy and loss comparisons. VER-Net and VGG19 both achieved the highest accuracy of 97.47% for training, but for testing, VER-Net emerged as the sole highest accuracy achiever with 91%. NASNetLarge got the lowest accuracy on both occasions, with 69.51% and 64% training and testing accuracy, respectively. Similar to accuracy, VER-Net and VGG19 both managed the lowest loss of 0.07% for training, and VER-Net was the sole lowest loss achiever with 0.34%. Here also, NASNetLarge performed worst on both occasions with 0.66% and 0.80% training and testing loss, respectively.

**Fig. 11:**
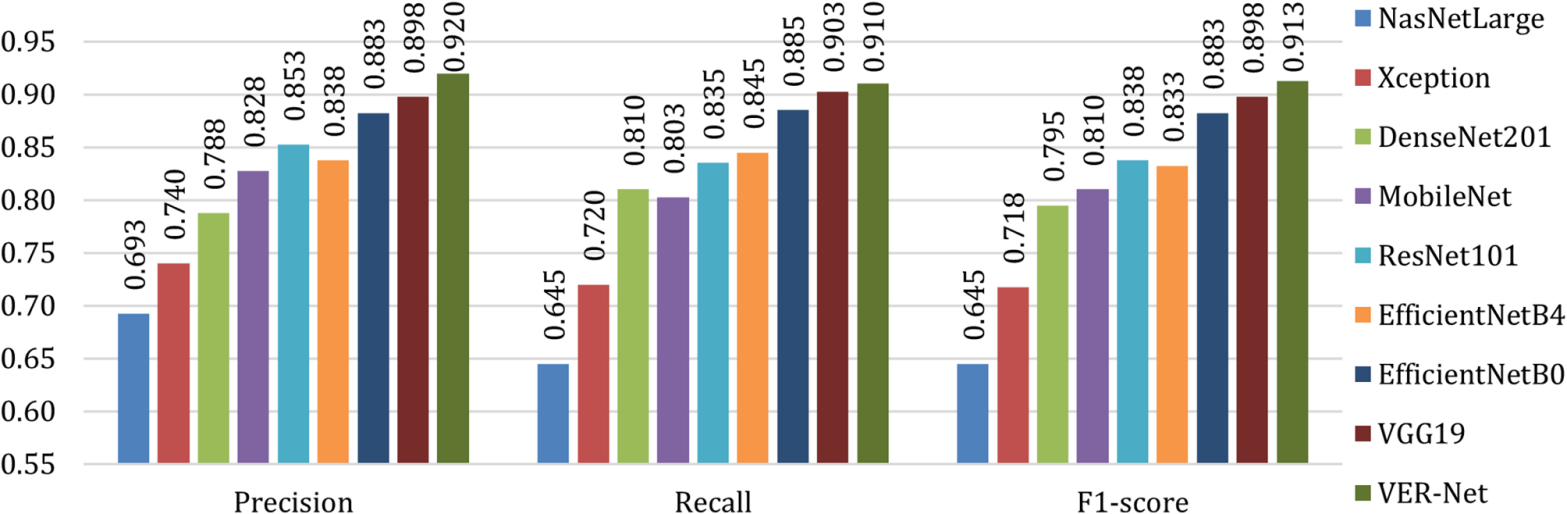
Macro average comparison of VER-Net and other models

Table 8 notes all classes’ precision, recall and F1-score values to compare VER-Net with other models. The macro average of these metrics for all four classes is shown in Fig. 11. For all three instances, i.e., precision, recall and F1-score, VER-Net outperformed with 0.920, 0.910, and 0.913, respectively. VGG19 and EficientNetB0 emerged as the second and third-best performers, whereas NASNetLarge was the worst performer with 0.693, 0.645, and 0.645 for precision, recall and F1-score, respectively.

**Fig. 12:**
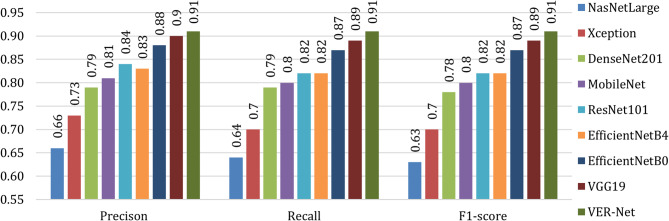
Weighted average comparison of VER-Net and other models

In Fig. 12, VER-Net is compared with others in terms of weighted average for precision, recall and F1-score. Here, we used a uniform weight of 1.5 for all classes. Like the macro average, VER-Net was the top performer for all three metrics, followed by VGG19 and EficientNetB0, and NasNetLarge was the worst performer. As shown in Table 8, NasNetLarge classifies the non-cancerous cells with 100% accuracy; in fact, it performs the best among all models but performs very poorly for the cancerous cells.

**Table 8:** Class-wise values of precision, recall and F1-score for VER-Net and other models

Model	Class	Precision	Recall	F1-score
NasNetLarge	Adenocarcinoma	0.49	0.76	0.59
	Large cell carcinoma	0.62	0.32	0.42
	Normal	1	1	1
	Squamous cell carcinoma	0.66	0.5	0.57
Xception	Adenocarcinoma	0.62	0.78	0.69
	Large cell carcinoma	0.56	0.7	0.63
	Normal	1	0.93	0.96
	Squamous cell carcinoma	0.78	0.47	0.59
DenseNet201	Adenocarcinoma	0.79	0.62	0.70
	Large cell carcinoma	0.73	0.93	0.82
	Normal	0.81	0.93	0.87
	Squamous cell carcinoma	0.82	0.76	0.79
MobileNet	Adenocarcinoma	0.66	0.86	0.75
	Large cell carcinoma	0.80	0.67	0.73
	Normal	1	0.98	0.99
	Squamous cell carcinoma	0.85	0.70	0.77
ResNet101	Adenocarcinoma	0.70	0.86	0.77
	Large cell carcinoma	0.85	0.80	0.82
	Normal	1	0.98	0.99
	Squamous cell carcinoma	0.86	0.70	0.77
EfficientNetB4	Adenocarcinoma	0.74	0.82	0.78
	Large cell carcinoma	0.76	0.95	0.84
	Normal	0.97	0.98	0.97
	Squamous cell carcinoma	0.88	0.63	0.74
EfficientNetB0	Adenocarcinoma	0.88	0.77	0.82
	Large cell carcinoma	0.83	0.96	0.89
	Normal	1	0.95	0.98
	Squamous cell carcinoma	0.82	0.86	0.84
VGG19	Adenocarcinoma	0.91	0.79	0.85
	Large cell carcinoma	0.78	0.96	0.86
	Normal	1	0.95	0.98
	Squamous cell carcinoma	0.90	0.91	0.90
VER-Net	Adenocarcinoma	0.81	0.93	0.87
	Large cell carcinoma	0.91	0.96	0.93
	Normal	1	0.93	0.96
	Squamous cell carcinoma	0.96	0.82	0.89

**Fig. 13:**
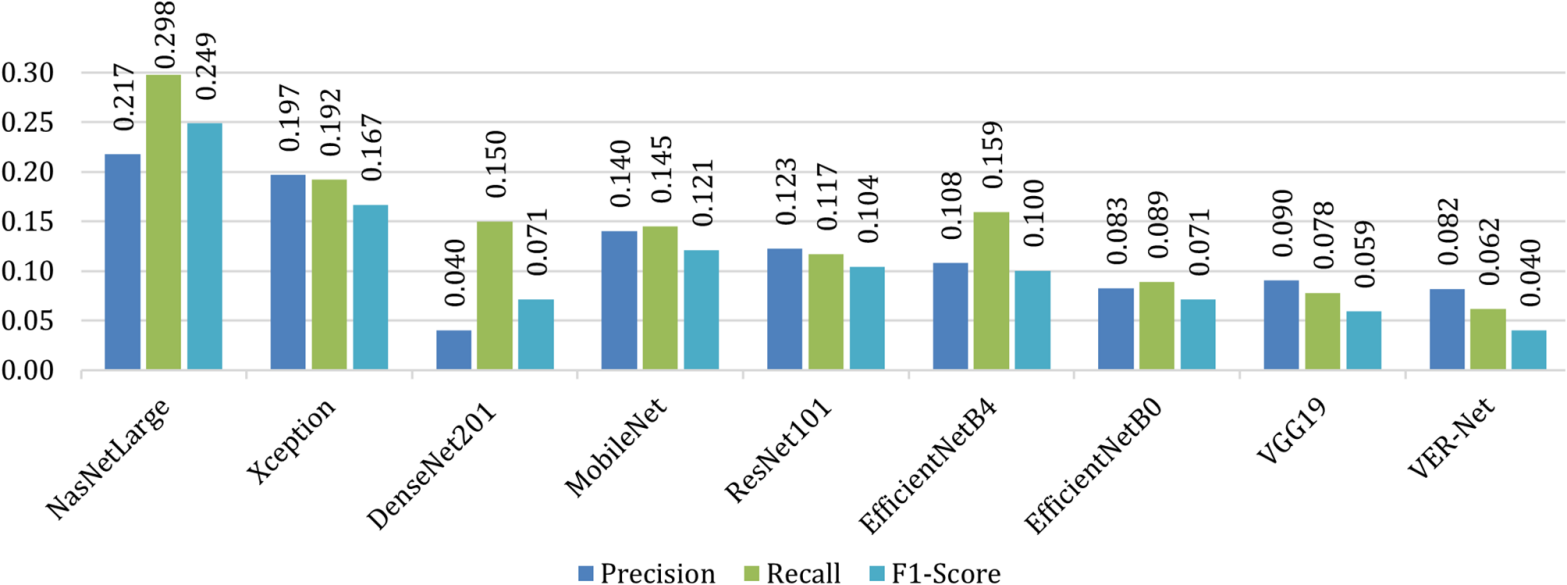
Standard deviation for precision, recall and F1-score of all classes

To assess the performance variations of VER-Net, we calculated the standard deviation to calculate the mean-variance across the classes for precision, recall and F1-score. A lower value suggests that the model is effective for all classes equally. In contrast, a higher variation suggests bias to a certain class. From Fig. 13, it can be observed that VER-Net has the lowest variations for recall and F1-score of 0.062 and 0.04, respectively. However, as an exception in the case of precision, VER-Net is bettered by DenseNet201 with a margin of 0.042 variations. This can be reasoned as VER-Net attained 100% precision for the Normal class. Nevertheless, VER-Net has significantly lower variance across three metrics than DenseNet201.

#### Comparing VER-Net with literature

In the previous section, we established the superiority of VER-Net over other established transfer learning models. To prove the ascendency of VER-Net further, we compared it with the results of some similar recent experiments, available in the literature pertaining to detecting lung cancer based on CT scan images using transfer learning methods. A comparative summary is given in Table 9.

#### Discussion

The above experiments and results clearly show that the proposed VER-NET performed well in detecting lung cancer in most of the performance testing. It is the overall best performer among the nine transfer learning models. One of the reasons for this is that we incorporated the best three models (considered in this experiment) into the VER-NET. Besides, we optimally designed the VER-NET architecture for its best performance. Furthermore, to make the model more generalized, we generated additional synthetic lung cancer images in addition to the original image dataset.

To balance the dataset, we performed image augmentation, which might make slight changes in the real images. So, the performance of VER-Net might vary little on a balanced real dataset where there is no need for synthetic augmentation. The images were generated with 64 × 64 pixels, which is insufficient for the analysis of medical images. For cancer cell detection based on cell images, high-resolution images are crucial.

Since VER-Net is an ensembled model comprising three transfer learning, it is obvious that it should increase the computational complexity, requiring longer for training. However, this should not be a discouraging factor in a lifesaving application like cancer detection, where accuracy and precision matter most.

**Table 9:** Comparing VER-Net with recent literature

Ref	Model	Dataset	Accuracy
Wang et al. [28]	AlexNet, VGG16, DenseNet, and DRNN	CT images of lung cancers collected from Shandong Provincial Hospital	85.71% with DRNN
Han et al. [29]	Ten machine learning models and VGG16	Department of Nuclear Medicine, Peking University cancer hospital	84.10% with VGG16
Nóbrega et al. [31]	VGG16, VGG19, MobileNet, Xception, InceptionV3, ResNet50, Inception-ResNet-V2, DenseNet169, DenseNet201, NASNetMobile, NASNetLarge, multilayer perceptron, SVM, k-nearest neighbors, and random forest.	LIDC/IDRI	88.41% with CNN-ResNet50 + SVM-RBF
Worku et al. [33]	Denoising first two-path CNN (DFD-Net)	Kaggle Data Science Bowl 2017 challenge (KDSB) and LUNA 16	87.80%
Chon et al. [45]	Linear, Vanilla 3D CNN, and 3D GoogLeNet	KDSB	75.10% with GoogLeNet
This paper	VER-Net (VGG19 + EfficientNetB0 + ResNet101)	Kaggle chest CT images	91.00%

## Conclusions and future scope

Incorporating transfer learning into lung cancer detection models has shown improved performance and robustness in various studies. In this paper, we concatenated three transfer learning models, namely, VGG19 + EfficientNetB0 + ResNet101, to build an ensembled VER-Net model to detect lung cancer. We used CT scan images as input to the model. To make VER-Net effective, we conducted data preprocessing and data augmentation. We compared the performance of VER-Net with eight other transfer learning models. The comparative results were assessed through various performance evaluation metrics. It was observed that VER-Net performed best in all metrics. VER-Net also exhibited better accuracy than similar empirical studies from the recent literature.

Here, we incorporated the three top-performing transfer models in the hybrid VER-Net architecture. Further experimentation can be done on this ensembling approach. For example, other models can be tried in different combinations. Also, transfer learning models of different families can be tried.

We plan to extend the use of the VER-Net model for identifying lung cancer where only chest X-ray images are available. Furthermore, this model can also be applied to assess the severity of lung cancer if the patient is already infested. Considering the success of VER-Net in detecting lung cancer, it can be used for other diseases where CT scan images are useful to identify the disease.

## Data Availability

No datasets were generated or analysed during the current study.

## Footnotes

1 https://www.who.int/news-room/fact-sheets/detail/cancer.

2 https://www.iarc.who.int/.

3 https://www.kaggle.com/datasets/mohamedhanyyy/chest-ctscan-images.

4 https://www.cancercenter.com/cancer-types/lung-cancer/types/adenocarcinoma-of-the-lung.

5 https://www.verywellhealth.com/large-cell-carcinoma-of-the-lungs-2249356.

6 https://www.mayoclinic.org/diseases-conditions/squamous-cell-carcinoma/.
