# Correction: VER-Net: a hybrid transfer learning model for lung cancer detection using CT scan images

**DOI:** 10.1186/s12880-024-01315-3

**Published:** 2024-05-31

**Authors:** Anindita Saha, Shahid Mohammad Ganie, Pijush Kanti Dutta Pramanik, Rakesh Kumar Yadav, Saurav Mallik, Zhongming Zhao

**Affiliations:** 1 Department of Computing Science and Engineering, IFTM University, Moradabad, Uttar Pradesh India; 2 AI Research Centre, Department of Analytics, School of Business, Woxsen University, Hyderabad, Telangana 502345 India; 3 School of Computer Applications and Technology, Galgotias University, Greater Noida, Uttar Pradesh 203201 India; 4 Department of Computer Science & Engineering, MSOET, Maharishi University of Information Technology, Lucknow, Uttar Pradesh India; 5 Department of Environmental Health, Harvard T. H. Chan School of Public Health, Boston, MA USA; 6 Center for Precision Health, McWilliams School of Biomedical Informatics, The University of Texas Health Science Center at Houston, Houston, TX 77030 USA

**Correction to: Saha et al. BMC Medical Imaging (2024) 24:120**.

10.1186/s12880-024-01238-z.

Due to a typesetting error, the last page of the PDF version of the Original Article was cut. Thus, reference number 12–45 were not shown.

Reference 12–45 are as follows:

The original article has been corrected.
